# Characterization of *Prdm9* in Equids and Sterility in Mules

**DOI:** 10.1371/journal.pone.0061746

**Published:** 2013-04-22

**Authors:** Cynthia C. Steiner, Oliver A. Ryder

**Affiliations:** Genetics Division, San Diego Zoo Institute for Conservation Research, San Diego Zoo Global, Escondido, California, United States of America; Virginia Tech, United States of America

## Abstract

*Prdm9* (*Meisetz)* is the first speciation gene discovered in vertebrates conferring reproductive isolation. This locus encodes a meiosis-specific histone H3 methyltransferase that specifies meiotic recombination hotspots during gametogenesis. Allelic differences in *Prdm9*, characterized for a variable number of zinc finger (ZF) domains, have been associated with hybrid sterility in male house mice via spermatogenic failure at the pachytene stage. The mule, a classic example of hybrid sterility in mammals also exhibits a similar spermatogenesis breakdown, making *Prdm9* an interesting candidate to evaluate in equine hybrids. In this study, we characterized the *Prdm9* gene in all species of equids by analyzing sequence variation of the ZF domains and estimating positive selection. We also evaluated the role of *Prdm9* in hybrid sterility by assessing allelic differences of ZF domains in equine hybrids. We found remarkable variation in the sequence and number of ZF domains among equid species, ranging from five domains in the Tibetan kiang and Asiatic wild ass, to 14 in the Grevy’s zebra. Positive selection was detected in all species at amino acid sites known to be associated with DNA-binding specificity of ZF domains in mice and humans. Equine hybrids, in particular a quartet pedigree composed of a fertile mule showed a mosaic of sequences and number of ZF domains suggesting that *Prdm9* variation does not seem by itself to contribute to equine hybrid sterility.

## Introduction

A fascinating unresolved question in evolutionary biology focuses on mechanisms of formation of new species and the processes that preserve species as separate entities. Species maintain their integrity through a set of premating and postmating reproductive barriers. Among them, hybrid sterility is one of the earliest postzygotic isolating barriers in species crosses, generally occurring in the heterogametic sex (XY or ZW), a pattern referred to as Haldane’s rule [1]. Postzygotic reproductive barriers are thought to occur through the acquisition of genetic incompatibilities in divergent populations, as proposed by the Dobzhansky-Muller model [2], in which negative epistasis between alleles/loci produces sterility or inviability of hybrids [3].

Few genes have been associated with hybrid sterility in model species [4], [5]. In *Drosophila*, some examples include the *Odysseus-site homeobox* [6], *JYAlpha* [7] and *Overdrive* [8] genes. More recently, *Prdm9* (*Meisetz)* has been identified as the first speciation gene conferring reproductive isolation in vertebrates [9]. This gene encodes a meiosis-specific histone H3 methyltransferase that is only expressed in germ cells entering the meiotic prophase [10], [11]. *Prdm9* contains KRAB and SET domains that are highly conserved among metazoans [12], followed by a set of Cys_2_His_2_ repeated zinc finger (ZF) domains in tandem near the carboxy-terminal region [13]. The ZF domains are the DNA-binding regions that confer specificity to the gene in a modular manner. The structure of ZF domains is well established, consisting of 21 residues of conserved amino acids that coordinate and position a highly variable nucleotide contact region, and seven conserved amino acid linkers that join adjacent zinc fingers [14]. *Prdm9* ZF domains exhibit rapid evolution and positive selection in a variety of organisms including humans, mice, cattle and salmon [12], [15]. In other species however, this gene is absent (birds, lizards, snakes) or has acquired disrupting mutations, such as a pseudogene in dogs [16].

*Prdm9* in vertebrates is responsible for activating and specifying genome-wide meiotic recombination hotspots [17]–[21]. *Prdm9*-null mice display arrest of gametogenesis in meiotic prophase I and impaired double-strand break repair [10]. Allelic differences in the number of ZF domains in *Prdm9* have been associated to hybrid sterility in house mouse crosses due to failure of spermatogenesis. For instance, an allele of *Prdm9* encoding 13 ZF domains (PRDM9^B6^) in *Mus musculus domesticus* (Mmd) causes sterility in F1 hybrid males when combined with another Mmd allele containing 14 ZF domains (PRDM9^C3H^) [9]. Furthermore, in humans, rare dominant non-synonymous mutations in *Prdm9* are associated with azoospermia also suggesting the presence of allelic incompatibilities [22].

Mules, hybrid offspring of a female horse and a male donkey, also exhibit a similar spermatogenesis breakdown at the pachytene stage of meiosis [23], [24], making *Prdm9* an interesting candidate gene for evaluation in interspecific equine crosses. Extant equids belong to a recently-evolved group of mammals diverging from a common ancestor about four million years ago [25], [26]. Due to a relatively recent divergence time, many viable but typically infertile equid hybrid combinations can be produced, not only via human-mediated reproduction but naturally [27], [28]. Differences in the number and structure of the haploid sets of chromosomes have been argued to form the basis for the inability of chromosome pairs to synapse in meiosis, producing sterility in equine hybrids [29]. However, reported instances of female mules and other equine hybrids with odd chromosome number given birth to perfectly viable offspring [30]–[33], suggest that mechanisms other than chromosomal differences contribute to hybrid sterility in equids.

In this study, we investigate the role of the *Prdm9* gene in equine hybrid sterility by assessing allelic differences of ZF domains in hybrids, including a fertile mule pedigree. For that purpose, we characterized *Prdm9* in all species of equids by determining patterns of sequence variation of ZF domains, and estimating positive selection. While this approach identified radical alterations in the number of ZF domains and rapid evolution among species, *Prdm9* allelic variation does not seem by itself to produce sterility in equids.

## Methods

### Ethics Statement

All samples were collected from postmortem animals or opportunistically during medical examination, according to the IACUC number 12-023. This study was approved by the Zoological Society of San Diego, Institutional Animal Care and Use Committee (N.H.I Assurance A3675-01).

### Sampling

We studied a total of 14 equid individuals belonging to eight species: domestic horse, *Equus caballus* (2 individuals), Przewalski’s horse, *E. przewalskii* (2), the Tibetan kiang, *E. kiang* (1), Asiatic wild ass, *E. hemionus* with two subspecies (onager and kulan; 3), African wild ass, *E. asinus* (2), mountain zebra, *E. zebra* (2), Burchell’s zebra, *E. burchelli* (1), and Grevy’s zebra, *E. grevyi* (1). Additionally, seven equine hybrids were also examined corresponding with: *E. hemionus kulan* × *E. kiang*, *E. przewalskii* × *E. caballus*, *E. grevyi* × *E. caballus*, *E. hemionus kulan* × *E. asinus*, a fertile mule (*E. asinus* × *E. caballus*) and her two offspring (*E. asinus* × mule). DNA extractions were performed from blood, testis, spleen and skin biopsies (Table S1) using the phenol/chloroform method or DNeasy blood and tissue kit (Qiagen). DNA samples are banked at the San Diego Zoo Institute for Conservation Research.

### Equid *Prdm9* Sequences

Well annotated *Prdm9* sequences from human and mouse were obtained from NCBI, Ensembl, and UCSC browsers. These sequences were used to interrogate the domestic horse genome (Sep.2007 Broad/equCab2) and predict the *Prdm9* gene using TBLASTn. Best hits showed sequence identities higher than 75% for all comparisons (horse-mouse, horse-human). The horse DNA sequence was pulled out and used for designing primers in conserved regions of the alignment of mouse, human and horse sequences. Specifically, primers were designed to amplify the final exon of *Prdm9* in equids containing the ZF domains: *Prdm9*_I5F 5′CAGGCAGCCTTGTCAACATCTACCCT, *Prdm9*_I6Rb 5′ CGTTGGAGCTGGAGTATGGAGT, and *Prdm9*_IF 5′ GAGGCTTCAATGACAGGGCAAGTCTTAT.

Polymerase chain reaction (PCR) amplifications were performed in a 20 µl volume using Eppendorf Mastercycler Gradient thermal cyclers. Each reaction included 30 ng of template DNA, 10 µl of 1X Taq buffer with 1.5 mM MgCl_2_ (Applied Biosystems), 0.3 µl of 10 mM deoxynucleoside triphosphates, 0.6 µl lM of each primer, and 0.15 units Taq DNA polymerase (Applied Biosystems). The PCR cycling conditions were 95°C for 6 min, followed by 34 cycles of denaturation at 94°C for 1 min, annealing at 59–60°C for 1 min, and 72°C extension for 1 min, with a final extension at 72°C for 10 min.

All PCR products were gel purified using Qiaquick gel purification kit (Qiagen), and cloned using the TOPO TA cloning kit (Invitrogen) following the manufacturer’s instructions. Cloning was implemented due to detection of two PCR products of similar size on 2% agarose gels in some equid species and hybrids. M13 forward and reverse primers plus *Prdm9* primers were used to sequence a minimum of five positive clones per species and hybrid. DNA sequences were edited and aligned with Sequencher 3.1.1 (Gene Codes, Ann Arbor, MI) and Geneious v1.2.1 [34], and then adjusted by eye, conserving the ZF domain reading frame. All equid sequences were translated and tested for encoding multiple ZF domains in-frame. Equid *Prdm9* sequences were submitted to Genbank (accession numbers KC209783–KC209813).

### Equid *Prdm9* cDNA

Total RNA was extracted from Asiatic wild ass (onager) testis using a trizol reagent. RT-PCR was set up to select for poly (A) RNA (mRNA) with oligo (dT), according to the superscript III first-strand synthesis system (Invitrogen). Amplification of target cDNA was carried out using specific primers that were also employed for sequencing: *Prdm9*_E6F_cDNA 5′ AGCTAGAGATCCATCCATGTC, and *Prdm9*_I6R_cDNA 5′ GTCCTCTTGGGGCTGAGACGTGAT. Onager cDNA was sequenced to confirm the expression of *Prdm9* in equid testes, and validate *Prdm9* sequences obtained from genomic DNA. Sequences were edited and aligned as previously described.

### *Prdm9* Ortholog Identification and Character Mapping

Orthology of equid *Prdm9* sequences was initially verified using TBLASTn. In all cases, best hits (e <10^−6^) always corresponded to *Prdm9* sequences from other species (e.g., *Mus*, *Homo*, *Peromyscus*, *Apodemus*) with average sequence identity higher than 75%. Known *Prdm9* paralogs such as *Prdm7* never showed up as best hits. The highest-scoring *Prdm9* sequences from Genbank were taken for reciprocal best BLAST against equid sequences for validating putative orthologs. To verify sequence identity, equid *Prdm9* sequences were blasted against the domestic horse genome using BLAT (UCSC browser).

Presence/absence of ZF domains was mapped on a Bayesian phylogenetic tree of the family Equidae [35], using MacClade 4.08 [36] to verify patterns of ZF domain evolution among equid lineages. All characters were assumed to be unordered and equally weighted, and calculations were made considering only unambiguous changes.

### *dN/dS* Analyses

Due to the high degree of concerted evolution that may occur among ZF domains, pairwise analyses of the non-synonymous to synonymous rate ratio (dN/dS) of *Prdm9* sequences from different species may be mislead [15]. Considering that, positive selection analyses were performed only for ZF domains, comprising 28 amino acids with two cysteine and two histidine residues. ZF domains were compared within and among equid species. Codons were aligned by a ClustalW [37] protein alignment (default parameters). Phylogenetic trees from each alignment were constructed using maximum parsimony in PAUP* version 4.0b10 [38] and PhyML 3.0 [39]. Topologies were accepted if no major discordance was observed between methods, and trees were supported by 1000 bootstrap replicates (>80%) [40]. The codeml program from PAML [41] was used to identify significant differences in likelihood values between nearly neutral (model 7) and positive selection models with unconstrained omega (model 8) and omega constrained to 1.0 (8a). P-value thresholds were corrected for multiple tests (Bonferroni correction). Amino acid sites under positive selection and P-values were inferred using the Bayes-Empirical-Bayes dN/dS approach in the unconstrained model 8.

## Results

### Equid *Prdm9* Sequences and Positive Selection

**Figure 1:**
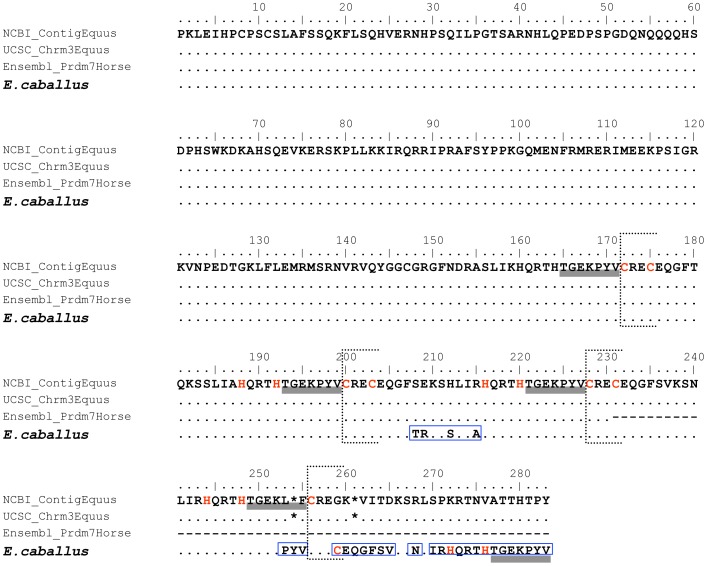
Alignment of the domestic horse *Prdm9* ZF domains. The sequence validated as *Prdm9* in the domestic horse (bold) was blasted against the horse genome using NCBI, UCSC and Ensemble browsers. Amino acid variation of the sequenced domestic horse is highlighted by blue open boxes. Linkers between ZF domains are indicated by grey bars, the beginning of ZF domains by dotted lines, stop codons by asterisks, and Cys_2_His_2_ residues by red letters.

We sequenced the final exon of *Prdm9,* which contains the ZF domains, in 14 equid individuals. These sequences ranged from 1074 bp to 1830 bp depending on the species. When blasted against the domestic horse genome, equid sequences matched an unannotated region in chromosome 3 (position 6378542–36379363) with 96% average identity. In particular, hits obtained from NCBI, UCSC, and Ensembl browsers were characterized by having early stop codons after the third ZF domain in comparison with the domestic horse sequence we generated (Figure 1), that showed a larger number of ZF domains. The UCSC and NCBI sequence hits were identical to a partial sequence from Ensembl annotated as *Prdm7*, a paralog gene of *Prdm9* found in other vertebrate species. This result suggests that equids may have at least two *Prdm* genes, one shorter copy annotated as *Prdm7* and a second copy not yet annotated corresponding to *Prdm9*, that differ in sequence and number of ZF domains.

**Figure 2:**
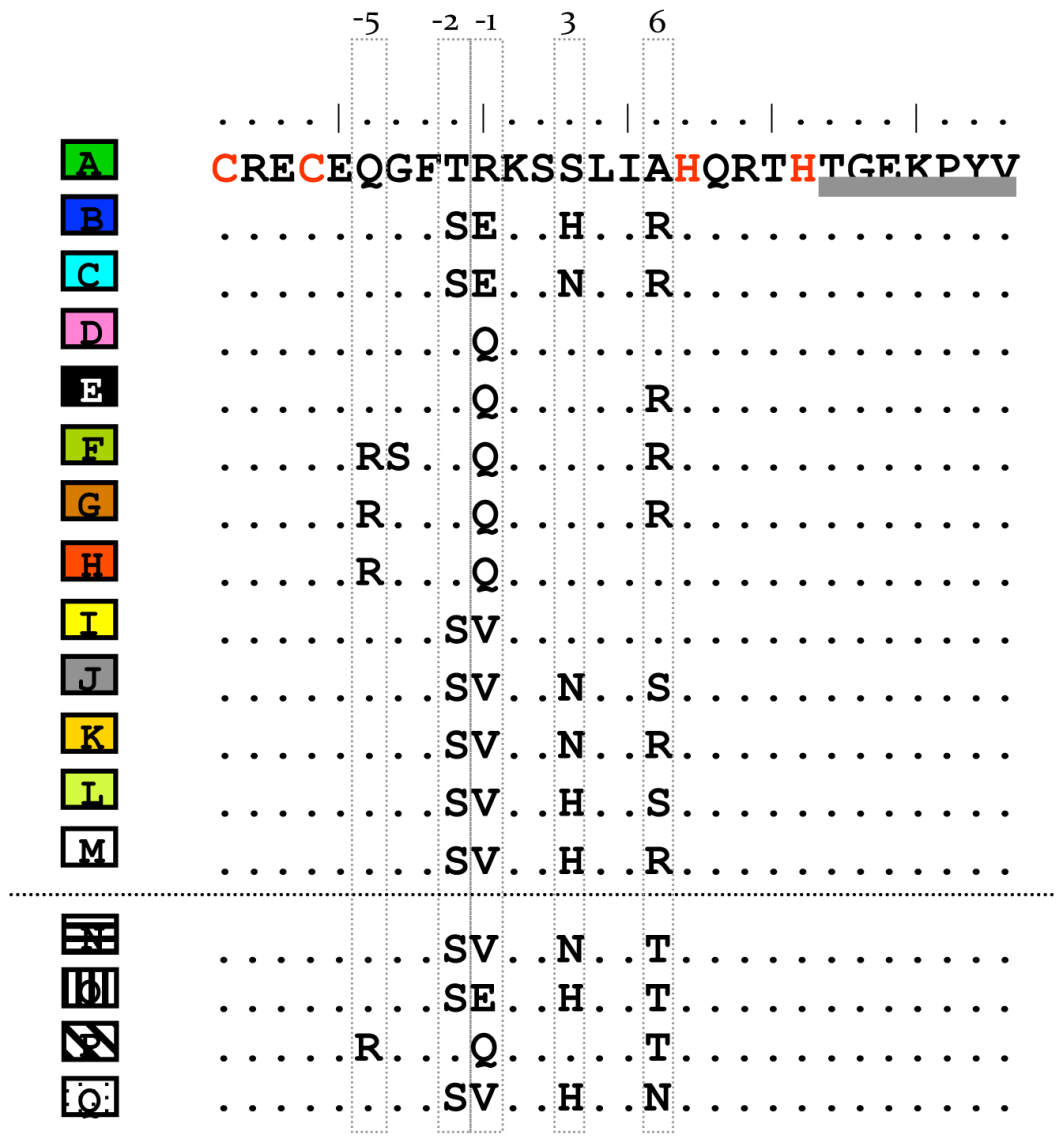
*Prdm9* ZF domains identified in equid species. Each ZF domain is color/letter coded and amino acid variation is indicated at positions −5, −2, −1, 3, and 6 (grey open boxes). ZF domains under the dotted lines are those only identified in equine hybrids but not in equid species. The seven amino acids linker is designated by grey bars and Cys_2_His_2_ residues by red letters.

**Figure 3:**
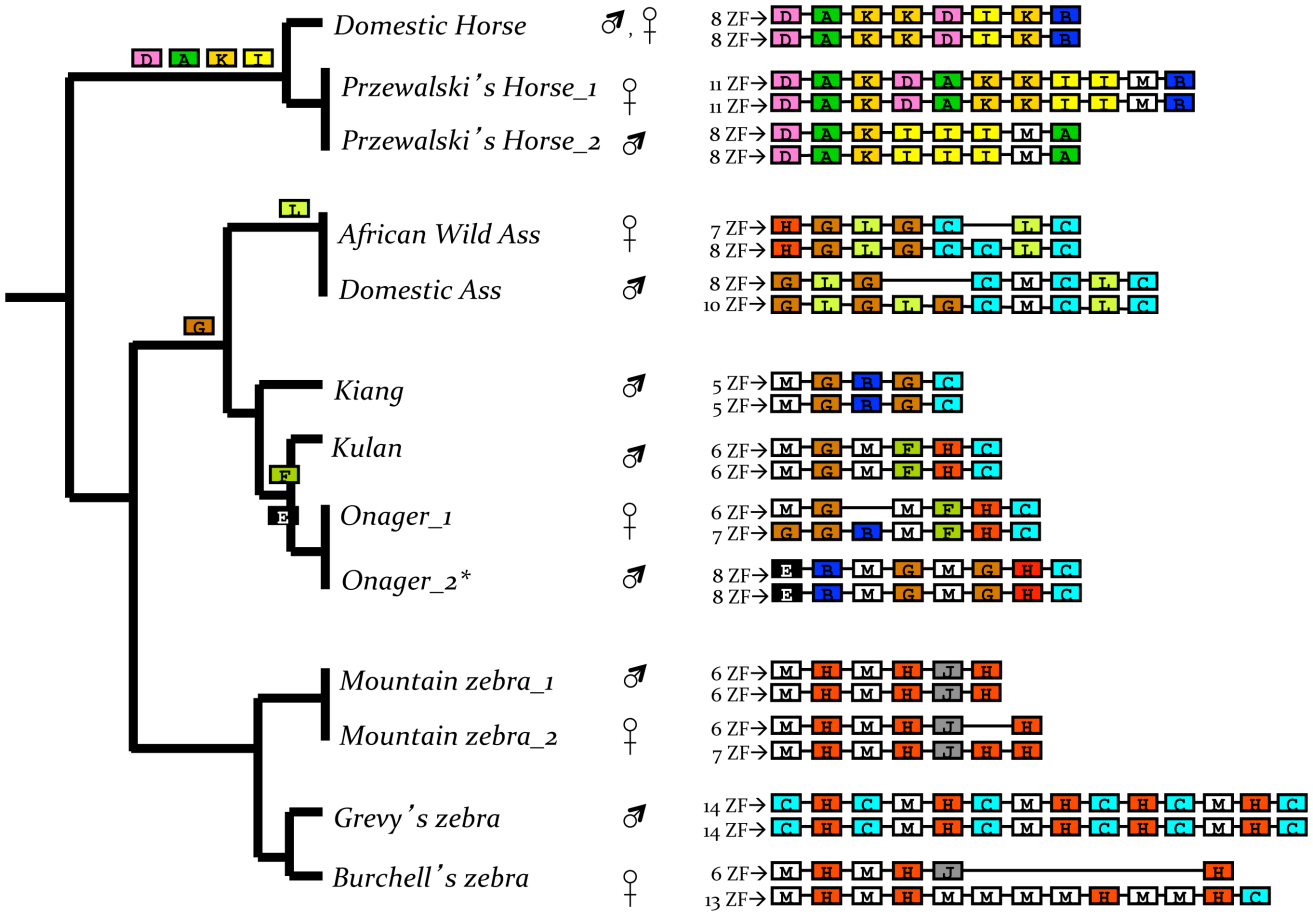
Diploid structure of *Prdm9* ZF domains in equids. Number and sequence of ZF domains are depicted, with individual’s gender shown. Color/letter coded ZF domains correspond to Figure 2. Lineage-specific ZF domains are mapped on the phylogeny of equids according to [33]. Domestic horse individuals showed the same *Prdm9* sequence for the last exon, thus only one sequence is depicted. The onager *Prdm9* sequence obtained from cDNA is designated by an asterisk.

In equid *Prdm9* sequences, we identified 13 different ZF domains characterized by amino acid variation at specific positions −5, −2, −1, 3 and 6 (Figure 2). All species showed a *Prdm9* final exon comprising a mixture of ZF domains, some appearing on species-specific lineages. For instance, ZF domains A, D, I and K were only identified in caballine horses, the lineage consisting of the domestic horse and Przewalski’s horse (Figure 3). Equids were highly variable in the number of ZF domains, ranging from five in the Tibetan kiang (*E. kiang*) to 14 ZF domains in the Grevy’s zebra (Figure 3). Intraspecific variation in the number of ZF domains also was observed in species such as the Przewalski’s horse, varying from 8 to 11, onager from 7 to 8, and mountain zebra from 6 to 7 ZF domains. Heterogeneity between chromosome pairs was found in the African wild ass, domestic ass, onager, and mountain zebra differing by one or two ZF domains, or up to seven in the Burchell’s zebra. Moreover, repeated ZF domains were recognized in all individuals examined suggesting that *Prdm9* may have undergone concerted evolution in equids; that is, some ZF domains have identical sequences, with intraspecific variation being less than that between species. In particular, Grevy’s zebra showed the highest number of identical repeated domains, with five copies of ZF domain H and six copies of C in both chromosomes.

**Figure 4:**
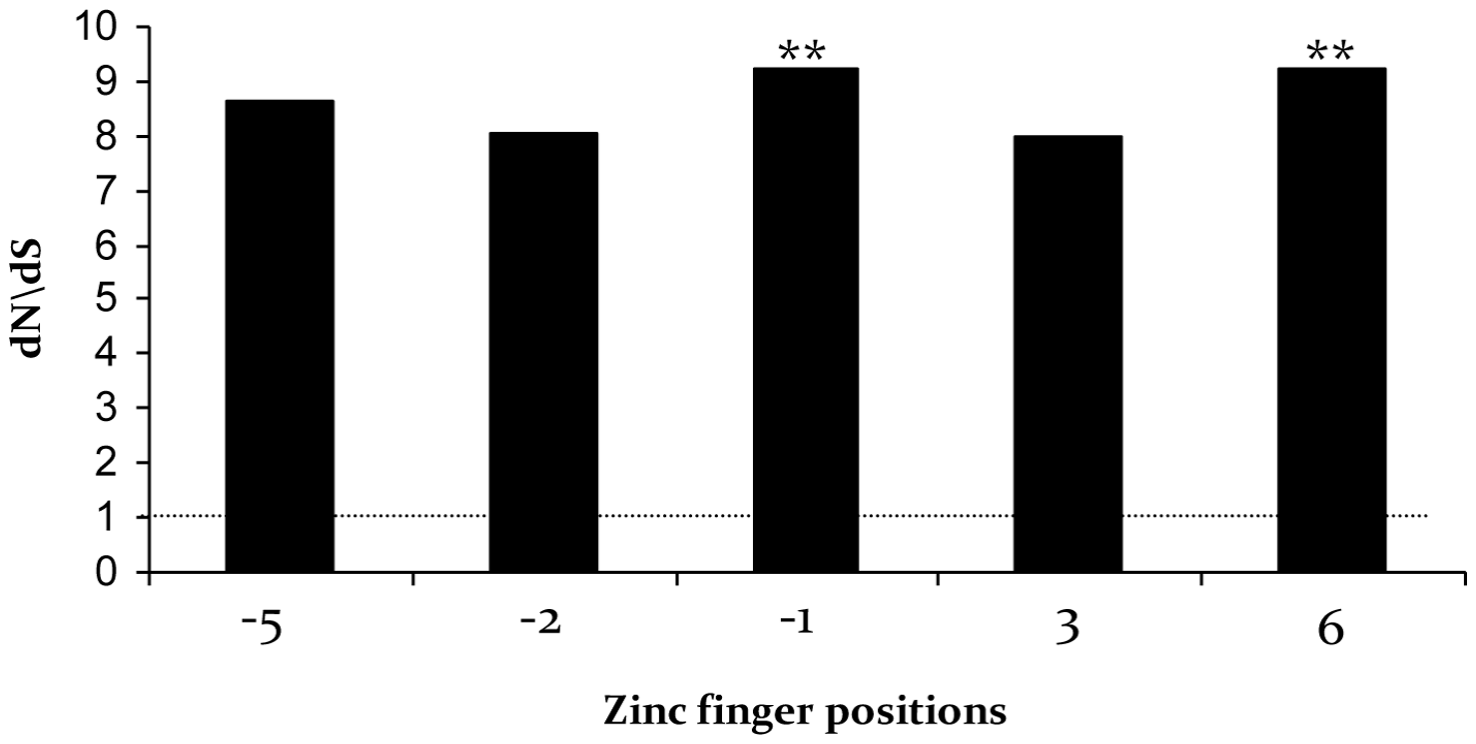
dN/dS estimates of variable amino acid sites of all equids. ZF domains of all species combined were tested for positive selection, with significant sites indicated by two asterisks (P<0.01).

Positive selection was evaluated in ZF domains of each species and species combined. The –2 δ ln*L* values between M8 and M8a models ranged from 7.35 (P = 0.0258) in the Asiatic wild ass to 15.05 (P = 0.0005) in the mountain zebra, suggesting the signature of positive selection in all species independently (P<0.05; Table S2). The Bayes-Empirical-Bayes dN/dS approach revealed positively selected sites corresponding with amino acid positions −5, −2, −1, 3 and 6 (Table 1). The analysis of all species combined also detected positive selection in ZF domains after correcting for multiple comparisons (P<0.0063). A smaller number of amino acid positions showed signature of positive selection, corresponding to sites −1 and 6, with dN/dS values surpassing the neutral rate, 8-fold or more (Figure 4).

**Table 1:** Positively-selected amino acid sites in the ZF domains of equids per species.

	Amino acid sites				
Species	−5	−2	−1	3	6
E. caballus		**	**	**	**
E. przewalskii		**	**	**	**
E. asinus		*	*	*	**
E. hemionus		*	*	*	*
E. kiang	*	**	**	**	
E. zebra	*	**	**	**	**
E. burchelli	**	**	**	**	**
E. grevyi	*	**	**	**	**

* Significant (P<0.05) and ** highly significant (P<0.01) positively-selected sites.

### *Prdm9* Sequence Variation in Equine Hybrids

**Figure 5:**
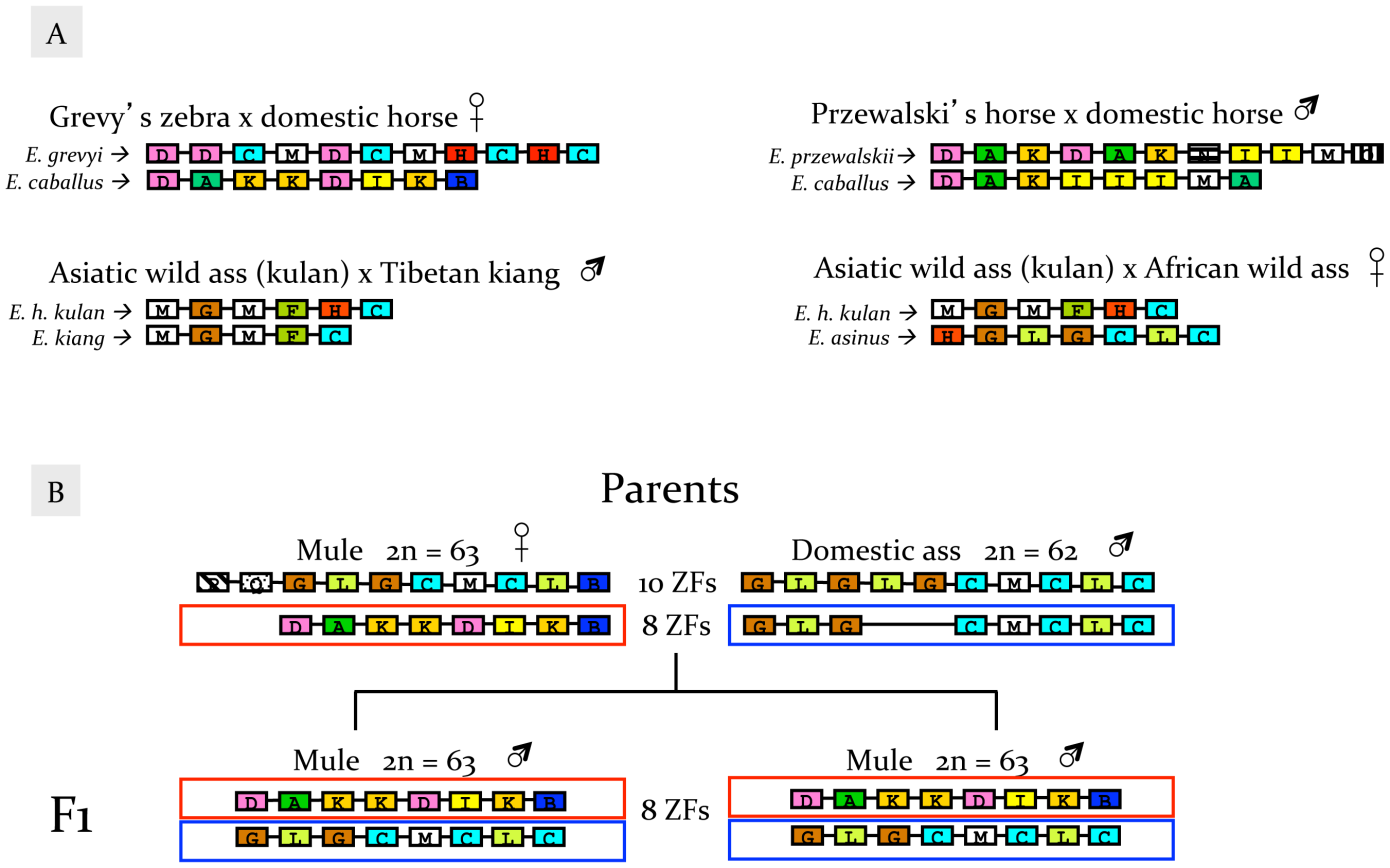
*Prdm9* ZF domains in equine hybrids. (A) Sequence variation and number of ZF domains in hybrids, with parental contribution inferred according to Figure 3. (B) Segregation of ZF domains in the pedigree of a fertile mule, with number and sequence of ZF domains indicated for parents (dam mule and sire donkey) and two offspring. Open red boxes indicate the chromosome received from the dam, and blue boxes from the sire. Diploid chromosome number (2n) and gender are shown for all individuals in the pedigree.

We also sequenced the last *Prdm9* exon of seven equine hybrids. Similar ZF domains were identified in hybrids relative to equid species. Some sequences were not detected, including ZF domains E and J. Four domains were noted only in the Przewalski’s horse × domestic horse hybrid and the fertile mule (Figure 2). All hybrids showed chimeric composition and variable number of ZF domains between chromosome pairs. *Prdm9* differed between hybrid chromosomes by a single domain in Asiatic wild ass (kulan) × Tibetan kiang (5 and 6 ZF) and Asiatic wild ass (kulan) × African wild ass (6 and 7), to three domains (8 and 11) in Grevy’s zebra × domestic horse and Przewalski’s horse × domestic horse hybrids (Figure 5A).

The reproductive status of the equine hybrids examined is unknown, except for one fertile female mule whose pedigree was available. We examined a quartet composed of the fertile mule (dam, 2n = 63), a donkey (sire, 2n = 62), and two F1 offspring (males, 2n = 63). We used this pedigree information for assessing the role of *Prdm9* allele differences in hybrid sterility of equids. Both parents were found to have the same number of ZF domains with one chromosome containing 10 and the other eight domains (Figure 5B). Sequences between chromosome pairs were similar in the donkey but chimeric in the dam mule, showing characteristics of domestic ass and horse. Parental chromosomes containing 8 ZF domains segregated to the F1 mules resulting in two chromosomes with equal number of ZF domains. F1 mules exhibited a chimeric composition of ZF domains as observed in the dam mule, and sequence variation at amino acid sites −2, −1, 3 and 6. These results suggest that neither the odd chromosome number (2n = 63) nor allelic differences in the number of ZF domains of *Prdm9* produce sterility in the female mule.

## Discussion

### Genetic Variation and Positive Selection

In this study, we generated partial sequences validated as *Prdm9* orthologs for 14 individuals of all equid species, and seven equine hybrids. Equid sequences diverged from the horse genome best hits in a manner suggesting that copies of *Prdm* genes have not yet been annotated in the horse genome. Duplication of *Prdm* genes is not unusual and has been described in other vertebrates, with at least 17 *Prdm* family members known in humans [42]. In primates, *Prdm7* is a known paralog of *Prdm9* that has undergone major structural rearrangements decreasing the number of encoded zinc fingers and modifying gene splicing [43]. Missing annotated *Prdm* genes in the horse genome may result from limitations associated with current genome assembling methods in identifying duplicated genomic regions or satellite DNA [44].

*Prdm9* ZF domains in equids showed dramatic numerical and amino acid composition variation. This high variation extends findings in other organisms demonstrating the rapid evolution of these domains [14], [15]. *Prdm9* ZF domains have been suggested to evolve rapidly due to the instability derived from the minisatellite structure of the ZF array [16]. Additionally, Oliver et al. [15] have speculated that *Prdm9* may function by binding to repetitive DNA sequences found at pericentric and centromeric regions, which are composed of rapidly evolving repetitive motifs [45]. *In vitro*, PRDM9 has shown to bind DNA at recombination hotspots via its ZF domains, and genetic manipulation of ZF domains changes the localization of hotspots [11], [18].

Multiple studies have suggested that *Prdm9* ZF domains have undergone strong positive selection, although this pattern may not encompass all metazoans [14], [15]. In rodents and primates, positive selection at specific DNA-binding positions (−1, 3, 6) has led to divergent evolution [15]. In equids, positive selection was restricted to amino acid positions −1 and 6 which correspond to sites responsible for determining DNA-binding specificity of *Prdm9*. Structural studies have shown that amino acids within the ZF α-helix at positions −1, 3 and 6 interact with bases 3, 2 and 1 respectively in the primary DNA strand [46]. Selection acting on residues −1 and 6 of the equid ZF domains may be altering DNA-binding preferences encoded by *Prdm9* among these species.

### Hybrid Sterility in Equids

Allelic incompatibilities in the *Prdm9* gene, as described in the house mouse [9], [47] represent a likely genetic mechanism to explain hybrid sterility in equids. However, in the fertile female mule, variation in the number of ZF domains does not seem to produce allelic incompatibilities within *Prdm9* and then sterility. This result is not unexpected considering that fertility of hybrid house mouse females is never compromised due to allelic differences of *Prdm9*, given that sterility is male-biased (heterogametic sex) in consistency with the Haldane’s rule [9].

No clear conclusion on the role of *Prdm9* in equine hybrid sterility can then be inferred based upon our results, other than that *Prdm9* variation of ZF domains does not seem by itself to contribute to sterility. This suggestion is confirmed by the single equid male known fertile in this study that also shows variation in the number of ZF domains, the domestic ass. *Prdm9* gene may still be an important speciation gene in equids via genetic incompatibilities with other loci (epistatic effects) [47]. In particular, *Prdm9* is known to interact with chromosome X (DXSr62 region) in the house mouse, and both loci are known to be necessary to produce F1 sterility [48]. Minor QTLs on chromosomes 13 and 14 also play a role in hybrid sterility, being sufficient to activate genetic incompatibilities in male mouse hybrids [48]. Hybrid sterility in the house mouse seems thus to have an oligogenic nature, indicating the importance of considering *Prdm9* genetic interactions.

Undoubtedly, *Prdm9* is not the sole gene associated with hybrid sterility in vertebrates, as this gene is absent in many species such as *Xenopus*, *Anolis*, and *Gallus* [12] or has become a pseudogene as confirmed in dogs [16]. Nonetheless, our findings of rapid evolution of *Prdm9* ZF domains in addition to the known function of this gene in specifying meiotic recombination hotspots, support its important role in gametogenesis in equids. Further studies considering additional equid hybrids and investigating genetic interactions of *Prdm9* with other loci will contribute to clarify the role of this gene establishing reproductive isolation barriers in this threatened group of mammals.

## Supporting Information

Table S1 **List of species and hybrids examined in this study.**
(DOC)

Table S2 **Positive selection analyses (M8** ***versus*** **M8a models comparison) of ZF domains of equid species.**
(DOC)

## References

[1] Laurie C (1997) The weaker sex is heterogametic: 75 years of Haldane’s rule. Genetics 147: 937–951. 9383043 10.1093/genetics/147.3.937 PMC1208269

[2] Muller H (1942) Isolating mechanisms, evolution, and temperature. In: T D, editor. Biological Symposia. Lancaster, PA: Jaques Cattell Press. pp. 71–125.

[3] Coyne J, Orr H (2004) Speciation. Sunderland, MA: Sinauer Associates, Inc. 545 p.

[4] Presgraves D (2010) The molecular evolutionary basis of species formation. Nature Reviews Genetics 11: 175–180. 10.1038/nrg2718 20051985

[5] Johnson NA (2010) Hybrid incompatibility genes: remnants of a genomic battlefield? Trends in Genetics 26: 317–325. 20621759 10.1016/j.tig.2010.04.005

[6] Perez D, Wu C (1995) Further characterization of the Odysseus locus of hybrid sterility in Drosophila: one gene is not enough. Genetics 140: 201–206. 7635285 10.1093/genetics/140.1.201 PMC1206547

[7] Masly J, Jones C, Noor M, Locke J, Orr H (2006) Gene transposition as a cause of hybrid sterility in Drosophila. Science 313: 1448–1450. 16960009 10.1126/science.1128721

[8] Phadnis N, Orr H (2009) A single gene causes both male sterility and segregation distortion in Drosophila hybrids. Science 323: 376–379. 19074311 10.1126/science.1163934 PMC2628965

[9] Mihola O, Trachtulec Z, Vlcek C, Schimenti JC, Forejt J (2009) A Mouse speciation gene encodes a meiotic Histone H3 Methyltransferase. Science 323: 373–375. 19074312 10.1126/science.1163601

[10] Hayashi K, Yoshida K, Yasuhisa M (2005) A histone H3 methyltransferase controls epigenetic events required for meiotic prophase. Nature 438: 374–378. 16292313 10.1038/nature04112

[11] Grey C, Barthes P, Chauveau-Le Friec G, Langa F, Baudat F, et al (2011) Mouse PRDM9 DNA-binding specificity determines sites of Histone H3 Lysine 4 Trimethylation for initiation of meiotic recombination. PLoS Biology 9: e1001176. 22028627 10.1371/journal.pbio.1001176 PMC3196474

[12] Ponting CP (2011) What are the genomic drivers of the rapid evolution of PRDM9? Trends in Genetics 27: 165–171. 21388701 10.1016/j.tig.2011.02.001

[13] Hayashi K, Matsui Y (2006) Meisetz, a novel histone tri-methyltransferase, regulates meiosis-specific epigenesis. Cell Cycle 5: 615–620. 16582607 10.4161/cc.5.6.2572

[14] Thomas JH, Emerson RO, Shendure J (2009) Extraordinary molecular evolution in the PRDM9 fertility gene. PLoS One 4: e805. 10.1371/journal.pone.0008505 PMC2794550 20041164

[15] Oliver PL, Goodstadt L, Bayes JJ, Birtle Z, Roach KC, et al (2009) Accelerated evolution of the *Prdm9* speciation gene across diverse metazoan taxa. PLoS Genetics 5: e1000753. 19997497 10.1371/journal.pgen.1000753 PMC2779102

[16] Munoz-Fuentes V, Di Rienzo A, Vila C (2011) *Prdm9*, a major determinant of meiotic recombination hotspots, is not functional in dogs and their wild relatives, wolves and coyotes. PLoS one 6: e25498. 22102853 10.1371/journal.pone.0025498 PMC3213085

[17] Myers S, Bowden R, Tumian A, Bontrop RE, Freeman C, et al (2009) Drive against hotspot motifs in primates implicates the PRDM9 gene in meiotic recombination. Science 327: 876–879. 20044541 10.1126/science.1182363 PMC3828505

[18] Baudat F (2010) PRDM9 is a major determinant of meiotic recombination hotspots in humans and mice. Science 328: 690–690. 20044539 10.1126/science.1183439 PMC4295902

[19] Parvanov ED, Petkov PM, Paigen K (2010) *Prdm9* controls activation of mammalian recombination hotspots. Science 327: 835–835. 20044538 10.1126/science.1181495 PMC2821451

[20] Berg IL, Neumann R, Sarbajna S, Odenthal-Hesse L, Butler NJ, et al (2011) Variants of the protein PRDM9 differentially regulate a set of human meiotic recombination hotspots highly active in African populations. Proceedings of the National Academy of Sciences of the United States of America 108: 12378–12383. 21750151 10.1073/pnas.1109531108 PMC3145720

[21] Segurel L, Leffler EM, Przeworski M (2011) The case of the fickle fingers: how the PRDM9 zinc finger protein specifies meiotic recombination hotspots in humans. PLoS Biology 9: e1001211. 22162947 10.1371/journal.pbio.1001211 PMC3232208

[22] Miyamoto T, Koh E, Sakugawa N, Sato H, Hayashi H, et al (2008) Two single nucleotide polymorphisms in PRDM9 (MEISETZ) gene may be a genetic risk factor for Japanese patients with azoospermia by meiotic arrest. Journal of Assisted Reproduction and Genetics 25: 553–557. 18941885 10.1007/s10815-008-9270-x PMC2593767

[23] Chandley AC, Jones RC, Dott HM, Allen WR, Short RV (1974) Meiosis in interspecific equine hybrids: the male mule and hinny. Cytogenetics and Cell Genetics 13: 330–341. 4430187 10.1159/000130284

[24] Horie T, Nishikawa Y (1959) Studies on the reproduction ability of mules II. On functions of testes. Bulletin of the National Institute of Agricultural Science Series G 8: 143–149.

[25] Lindsay E, Opdyke N, Johnson N (1980) Pliocene dispersal of the horse *Equus* and late Cenozoic mammalian dispersal events. Nature 287: 135–138.

[26] Hulbert RJ (1996) The ancestry of the horse. In: Olsen S, editor. Horses through time. Boulder, CO: Roberts Rinchart. pp. 11–34.

[27] Allen WR, Short RV (1997) Interspecific and extraspecific pregnancies in equids: anything goes. Journal of Heredity 88: 384–392. 9378914 10.1093/oxfordjournals.jhered.a023123

[28] Cordingley JE, Sundaresan S, Fischhoff I, Shapiro B, Ruskey J, et al (2009) Is the endangered Grevy’s zebra threatened by hybridization? Animal Conservation 12: 505–513.

[29] Benirschke K, Low RJ, Sullivan MM, Carter RM (1964) Chromosome study of an alleged fertile mare mule. Journal of Heredity 55: 31-. 10.1093/oxfordjournals.jhered.a107283 14134074

[30] Short RV, Chandley AC, Jones RC, Allen WR (1974) Meiosis in interspecific equine hybrids: The Przewalski horse/domestic horse hybrid. Cytogenetics and Cell Genetics 13: 465–478. 4462982 10.1159/000130300

[31] Ryder OA, Chemnlck LG, Bowling AT, Benirschke K (1985) Male mule foal qualifies as the offspring of a female mule and jack donkey. Journal of Heredity 76: 379–381. 4056372

[32] Rong R, Chandley AC, Song J, McBeath S, Tan PP, et al (1988) A fertile mule and hinny in China. Cytogenetics and Cell Genetics 47: 134–139. 3378453 10.1159/000132531

[33] Henry M, Gastal EL, Pinheiro LEL, Guimarmes SEF (1995) Mating pattern and chromosome analysis of a mule and her offspring. Biology of Reproduction Monograph Series 1: 273–279.

[34] Drummond A, Kearse M, Heled J, Moir R, Thierer T, et al. (2006) Geneious v1.2.1, Available: http://www.geneious.com/.

[35] Steiner C, Mitelberg A, Tursi R, Ryder O (2012) Molecular phylogeny of extant equids and effects of ancestral polymorphism in resolving species-level phylogenies. Molecular Phylogenetics and Evolution 65: 573–581. 22846684 10.1016/j.ympev.2012.07.010

[36] Maddison D, Maddison W (2005) MacClade 4.08. Sunderland, MA: Sinauer Associates, Inc.

[37] Thompson J, Higgins D, Gibson T (1994) CLUSTAL W: improving the sensitivity of progressive multiple sequence alignment through sequence weighting, position-specific gap penalties and weight matrix choice. Nucleic Acids Research 22: 4673–4680. 7984417 10.1093/nar/22.22.4673 PMC308517

[38] Swofford D (2002) PAUP*: Phylogenetic Analysis Using Parsimony (and Other Methods) 4.0 Beta. Sinauer Associates, Inc: Sunderland, MA.

[39] Guindon S, Dufayard JF, Lefort V, Anisimova M, Hordijk W, et al (2010) New Algorithms and Methods to Estimate Maximum-Likelihood Phylogenies: Assessing the Performance of PhyML 3.0. Systematic Biology 59: 307–321. 20525638 10.1093/sysbio/syq010

[40] Felsenstein J (1985) Confidence limits on phylogenies: An approach using the bootstrap. Evolution 39: 783–791. 28561359 10.1111/j.1558-5646.1985.tb00420.x

[41] Yang Z (1997) PAML: a program package for phylogenetic analysis by maximum likelihood. Computer Applications in the Biosciences 13: 555–556. 9367129 10.1093/bioinformatics/13.5.555

[42] Fog C, Galli G, Lund A (2011) PRDM proteins: Important players in differentiation and disease. BioEssays 34: 50–60. 22028065 10.1002/bies.201100107

[43] Fumasoni I, Meani N, Rambaldi D, Scafetta G, Alcalay M, et al (2007) Family expansion and gene rearrangements contributed to the functional specialization of PRDM genes in vertebrates. BMC Evolutionary Biology 7: 187. 17916234 10.1186/1471-2148-7-187 PMC2082429

[44] Alkan C, Sajjadian S, Eichler E (2011) Limitations of next-generation genome sequence assembly. Nature Methods 8: 61–65. 21102452 10.1038/nmeth.1527 PMC3115693

[45] Smith GP (1976) Evolution of repeated DNA sequences by unequal crossover. Science 191: 528–535. 1251186 10.1126/science.1251186

[46] Choo Y, Klug A (1994) Selection of DNA binding sites for zinc fingers using rationally randomized DNA reveals coded interactions. Proceedings of the National Academy of Sciences of the United States of America 91: 11168–11172. 7972028 10.1073/pnas.91.23.11168 PMC45188

[47] Flachs P, Mihola O, Šimeček P, Gregorová S, Schimenti JC, et al (2010) Interallelic and intergenic incompatibilities of the *Prdm9* (*Hst1*) gene in mouse hybrid sterility. PLoS Genetics 8(11): e1003044. 10.1371/journal.pgen.1003044 PMC3486856 23133405

[48] Dzur-Gejdosova M, Simecek P, Gregorova S, Bhattacharyya T, Forejt J (2012) Dissecting the genetic architecture of F1hybrid sterility in house mice. Evolution 66: 3321–3335. 23106700 10.1111/j.1558-5646.2012.01684.x

