# Supplementary material for: Characterization of Prdm9 in Equids and Sterility in Mules
Source: PLoS One. 2013 Apr 22;8(4):e61746. doi: 10.1371/journal.pone.0061746 (PMC3632555; doi:10.1371/journal.pone.0061746)
Supplement: Table S1 — List of species and hybrids examined in this study. (DOC) [file pone.0061746.s001.doc]

Table S1. List of species and hybrids examined in this study.

| **Species name** | **Common name** | **Sex** | **Species ID** | **Location*** | **Date of Collection** | **Sample type** |
| --- | --- | --- | --- | --- | --- | --- |
| *Equus asinus somalicus* | African wild ass | F | KB6132 | San Diego Zoo, SD | October, 1996 | -- |
| *Equus asinus* | Domestic ass | M | KB5101 | Imperial Ranch, NE | March, 1988 | Skin biopsy |
| *Equus caballus* | Domestic horse | M | OR535 | -- | -- | Testes |
| *Equus caballus* | Domestic horse | F | OR225 | -- | July, 1977 | Spleen |
| *Equus grevyi* | Grevy’s zebra | M | OR222 | San Diego Safari Park, SD | August, 1977 | Spleen |
| *Equus hemionus kulan* | Kulan | M | OR772 | San Diego Safari Park, SD | -- | -- |
| *Equus hemionus onager* | Onager | F | OR511 | San Diego Safari Park, SD | September, 1985 | -- |
| *Equus kiang* | Kiang | M | KB5908 | San Diego Safari Park, SD | October, 1986 | Skin biopsy |
| *Equus przewalskii* | Przewalski’s wild horse | M | OR758 | San Diego Safari Park, SD | -- | -- |
| *Equus przewalskii* | Przewalski’s wild horse | F | OR301 | San Diego Safari Park, SD | June, 1987 | Spleen |
| *Equus burchelli* | Burchell’s zebra | F | Kristina | -- | -- | -- |
| *Equus zebra* | Mountain zebra | F | Tamara | -- | -- | -- |
| *Equus zebra* | Mountain zebra | M | Solo | Canyon Colorado Equine Sanctuary, CO | November, 1989 | Blood |
|  |  |  |  |  |  |  |
| **Hybrids** |  |  |  |  |  |  |
| *E. asinus* x Mule | Mule offspring | M | KB6221 | Imperial Ranch, NE | March, 1988 | Skin biopsy |
| *E. asinus* x *E. caballus* | Mule | F | KB5099 | Imperial Ranch, NE | March, 1988 | Skin biopsy |
| *E. asinus* x Mule | Mule offspring | M | KB5100 | Imperial Ranch, NE | March, 1988 | Skin biopsy |
| *E. h. kulan* x *E. kiang* |  | M | KB7447 | Moscow Zoo, Russia | August, 1991 | -- |
| *E. przewalskii* x *E. caballus* |  | M | KB7903 | Askania-nova, Ukraine | October, 1991 | Skin biopsy |
| *E. grevyi* x *E. caballus* | Zorse | F | 1419 | -- | April, 1977 | Blood |
| *E. h. kulan* x *E. asinus* |  | F | 2823 | -- | April, 1979 | Blood |

KB and OR= San Diego Zoo species ID number

* Location when animal was sampled: SD= San Diego, NE= Nebraska, CO= Colorado
