# Supplementary material for: Characterization of Prdm9 in Equids and Sterility in Mules
Source: PLoS One. 2013 Apr 22;8(4):e61746. doi: 10.1371/journal.pone.0061746 (PMC3632555; doi:10.1371/journal.pone.0061746)
Supplement: Table S2 — Positive selection analyses (M8 versus M8a models comparison) of ZF domains of equid species. (DOC) [file pone.0061746.s002.doc]

Table S2. Positive selection analyses (M8 *versus* M8a models comparison) of ZF domains of equid species.

| **Species** | **ln*L*** | **ln*L*** | **– 2 δ ln*L*** | **P** |
| --- | --- | --- | --- | --- |
| *E. asinus* | -167.42 | -163.51 | **7.83** | **0.0200** |
| *E. burchelli* | -166.65 | -159.54 | **14.23** | **0.0008** |
| *E. caballus* | -168.87 | -162.70 | **12.34** | **0.0021** |
| *E. grevyi* | -156.13 | -151.05 | **10.16** | **0.0062** |
| *E. hemionus* | -173.52 | -169.84 | **7.36** | **0.0253** |
| *E. kiang* | -146.96 | -142.94 | **8.03** | **0.0181** |
| *E. przewalskii* | -170.26 | -164.14 | **12.23** | **0.0022** |
| *E. zebra* | -158.09 | -150.57 | **15.05** | **0.0005** |
| All combined | -219.11 | -211.51 | **15.20** | **0.0005** |

ln*L* and – 2 δ ln*L* = Likelihood values
